# Investigation of Swelling Behavior and Mechanical Properties of a pH-Sensitive Superporous Hydrogel Composite

**Published:** 2012

**Authors:** N. Vishal Gupta, H.G. Shivakumar

**Affiliations:** *Department of Pharmaceutics, JSS College of Pharmacy, JSS University, Sri Shivarathreeshwara Nagar, Mysore-570 015, Karnataka, India.*

**Keywords:** Superporous hydrogels, Composites, Radical polymerization, Swelling, Electron microscopy

## Abstract

The objective of the present study is to develop and investigate the swelling behavior of pH-sensitive Superporous Hydrogel (SPH) and SPH composite (SPHC). A novel superporous hydrogel containing poly (methacrylic acid-co-acrylamide) was synthesized from methacrylic acid and acrylamide through the aqueous solution polymerization, using N,N-methylenebisacrylamide as a crosslinker and ammonium persulfate as an initiator. SPHCs were made in the same way, except for the using of Ac-Di-Sol as a stabilizer. The synthesized SPH and SPHC were characterized by Fourier-transform infrared spectroscopy, swelling kinetics, porosity, mechanical properties and scanning electron microscopy. The swelling of SPH and SPHC was sensitive towards the pH, ionic strength, and temperature stimuli. The study of the surface morphology of SPH using scanning electron microscopy showed a highly porous structure. SPH polymers showed higher swelling ratio but less mechanical stability compared to SPHC polymers, which showed lower swelling ratio but a higher mechanical stability. With a change in pH from acidic to basic, a considerable increase in swelling was observed. Since the prepared SPH and SPHC swell only in the basic pH, it may be concluded that SPH and SPHC can be used as the pH-sensitive drug delivery system.

## Introduction

Hydrogels are three-dimensional crosslinked hydrophilic polymers that are able to swell in an aqueous environment without dissolution (1). Because of their high water affinity, environmental sensitivity and high permeability, hydrogels have been widely used as a carrier for drug delivery systems (2-4).

Stimuli-sensitive hydrogels (or smart hydrogels) are hydrogels that undergo large changes in the swelling ratio by only a small variation in environmental conditions, such as temperature (5), pH (6, 7), light (8), electric field (9, 10), pressure (11), carbohydrates (12) and antigens (13, 14). Among them, pH-sensitive hydrogels that change the properties depending upon the changes in pH have been extensively investigated for the development of new drug delivery systems (15). These gels can be prepared by the incorporation of one or more weakly acidic or basic monomers such as carboxylic acids and primary or substituted amines. Acidic gels are considered as good candidates for oral colon specific delivery of drugs that are susceptible to enzymatic degradation in the upper gastrointestinal tract. While these kinds of systems have slow equilibrium degree of swelling in acidic medium of stomach, their swelling degree increases as it passes down the gastrointestinal tract due to an increase in pH (16, 17). Although the swelling ratio changes greatly by a small change in an environmental factor, the kinetics of such changes has been slow. These hydrogels respond to the conditions in the order of hours to days, depending on their sizes. The response time has been shortened by reducing the dimensions of the hydrogels. Quite frequently, the diameter of particles or rods, and the thickness of sheets or membranes are brought to very small values to minimize the diffusion length of absorbed water. Dependence on the shape and size of these systems, however, limits their applications, especially when large structures are needed.

An alternative approach of making fast responsive hydrogel systems has been preparing the comb-type polymers. Comb-type polymers having hydrophobic pendant chains are known to collapse much quicker than conventional hydrogels (18). The increase in shrinking rate was, however, not associated with an increase in swelling rate.

Superporous hydrogels and their composites have been used for the development of gastroretentive devices, intestinal targeting of drugs and in fast dissolving tablets. Superporous hydrogels have also been developed and used as an appropriate carrier for the oral delivery of peptide and protein drugs (19, 20). Because of large numbers of interconnected pores that form the open channel structure, SPH and SPHC possess fast swelling and high swelling ratio (21, 22). Due to their high swelling ratio and carboxylic groups in their structure, these polymers have the capabilities of inhibiting proteolytic enzyme and opening the intercellular tight junctions that impede the absorption of hydrophilic macromolecular drugs. Besides, SPH and SPHC are safe excipients and do not cause any damage to the intestinal mucosal membranes and Caco-2 cell monolayers (23, 24).

In our previous work, we have synthesized poly (methacrylic acid-co-acrylamide) superporous hydrogels as pH-sensitive drug delivery systems for Pantoprazole Sodium (25). The objective of this study is to prepare and study the swelling behavior of superporous hydrogels and their composites employing acrylamide and methacrylic acid as monomers and methylene bisacrylamide as crosslinking agent. The composite material used was Ac-Di-Sol (cros-caramellose sodium). In order to improve the mechanical strength of the fully swollen SPH, SPHCs were prepared by adding Ac-Di-Sol during their synthesis. In this study, we have emphasized upon the high swelling capacities and mechanical properties with a focus on stimuli-sensitive swelling and water-retention capacities which were highly demanded when the polymers were developed as a potential drug delivery system. Therefore, the pH, salt and thermosensitive swelling of the SPH and SPHC were investigated in this study.

## Experimental

Acrylamide (AM) and Sodium bicarbonate were purchased from Loba Chemie, India. Methacrylic acid (MAA) was obtained from Aldrich, Germany. *N*,*N*-methylene-bis-acrylamide (BIS) and Pluronic-F127 (PF127) were supplied by Sigma Germany. Tetramethylethylenediamine (TEMED) and Ammonium persulphate (APS) were provided by Ranbaxy Fine Chemicals India. Ac-Di-Sol was a gift from Dr. Reddy’s Labs, India. MAA was distilled under reduced pressure before the use. All other reagents used were of analytical grade.

*Synthesis of superporous hydrogels*

**Figure 1:**
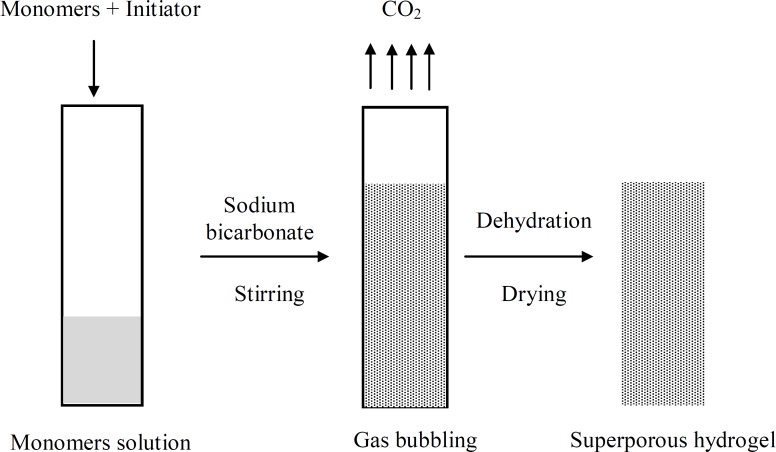
Diagrammatic representation of the general procedure for making superporous hydrogel

Superporous hydrogels were synthesized by the following procedure (25, 26). All ingredients except for sodium bicarbonate and Ac-Di-Sol were used as solution in distilled water. The following components were added sequentially into the glass test tube (with inner diameter of 16 mm and height of 100 mm) at ambient temperature: 300 µL of 50% AM, 150 µL of 50% MAA, 40 µL of 2.5% BIS, 30 µL of 10% PF127, 20 µL of 20% APS and 20 µL of 20% TEMED. The test tube was extensively mixed after adding each component. The pH was adjusted to 5.0 using sodium hydroxide solution. After 120 s, 100 mg of Sodium bicarbonate was added to the mixture, and the tube was vortexed. Polymerization was allowed to continue for 30 min at room temperature. The synthesis of SPHCs was similar to SPH. Ac-Di-Sol was added to the monomer mixture after adding APS and before adding TEMED. Three different amounts of Ac-Di-Sol, *i.e*. 100, 200 and 400 mg were used and the composites were designated as SPHC_100, _SPHC_200 _and SPHC_400_. The synthesized polymers were dialyzed against distilled water for 4 days in order to remove the unreacted components. The polymers were further dialyzed and dehydrated against absolute ethanol for 2 days. Thereafter, the polymers were put in an oven at 60^o^C for 24 h for complete drying. They were then cut into 5 mm thick circular discs and used for further studies. The synthesis procedure is diagrammatically represented in Figure 1.

*Swelling studies*

A completely dry, pre-weighed disc-shaped SPH and SPHC was weighed and then immersed in excess of swelling medium. At various time intervals, the hydrogel was removed from the solution and weighed after excessive solution on the surface was blotted. Data presented in this experiment was the mean values of triplicate measurements. Results were calculated according to the following equation:

Q = (M_s_ – M_d_) / M_d_

Here, *Q *is the swelling ratio, M_s_ is the mass in the swollen state and M_d_ is the mass in the dried state (27).

Five NaCl solutions (pH = 7*.*4) with different ionic strengths (0.0001-1 M) were used to evaluate the salt effect on the swelling of SPHC_100_. Different salt solutions (NaCl, CaCl_2_ and AlCl_3, _pH = 7.4, ionic strength = 0.01 M) were also used for evaluating the ionic effect. To study the pH sensitivity of the SPHC_100_, HCl or NaOH solutions with defined pH of 1.0, 2.0, 3.0, 4.9, 6.2 and 7.4 was used. To determine the effect of temperature on the swelling kinetics of SPHC_100_, deionized water at 20, 30, and 40^o^C was used as the swelling media.

Pulsatile pH-dependent swelling of the SPH and SPHC_100_ was evaluated at 37°C with alternation of swelling medium between the HCl buffer solution (pH = 1.2) and phosphate buffered solution (PBS, pH 7.4). The hydrogels were first swollen in HCl solution (pH = 1.2) for 30 min. The swollen hydrogels in the HCl solution were weighed at each given time and transferred to the PBS buffer. The same procedures were performed for swelling in PBS before the swollen hydrogels were transferred back to the HCl solution. The hydrogels were transferred to the alternating solutions every 30 min (28).

*Porosity measurement*

For porosity measurement, the solvent replacement method was used. Dried hydrogels were immersed in absolute ethanol overnight and weighed after that the excess ethanol on the surface was blotted. The porosity was calculated from the following equation:

Porosity = (M_2_ – M_1_) / ρV

Here, M_1_ and M_2_ are the mass of hydrogel before and after the immersion in absolute ethanol, respectively; ρ is the density of absolute ethanol and V is the volume of the hydrogel (29).

*Determination of void fraction*

The void fraction was calculated by the following equation:

Void Fraction = Dimensional volume of the hydrogel / Total volume of pores

The void fractions inside SPHs and SPHCs were determined by immersing the hydrogels in PBS upto equilibrium swelling. The dimensions of the swollen hydrogels were measured and by using these data, sample volumes were determined as the dimensional volume. In the meantime, the amount of absorbed PBS into the hydrogels was determined by subtracting the weight of dried hydrogel from the weight of swollen hydrogel. This value is given as the total volume of pores in the hydrogels (30).

*Water retention*

The following equation was used to determine the water retention capacity (WR_t_) as a function of time:

WR_t _= (W_p _- W_d_) / (W_s _- W_d_)

Here, W_d_ is the weight of the dried hydrogel, W_s_ is the weight of the fully swollen hydrogel, and W_p_ is the weight of the hydrogel at various exposure times. For the investigation of the water-retention capacity as a function of the time of exposure at 37°C of the hydrogels, the water loss of the fully swollen polymer at timed intervals was determined through gravimetry (31).

*Mechanical properties*

The compressive strengths of SPH and SPHC were determined using a bench comparator. Briefly, after that the fully swollen hydrogel was put longitudinally under the lower touch of a bench comparator, different scale loads were successively applied on the upper touch until a point was reached when the hydrogel could not support any more weight and completely fractured.

The pressure at that point was defined as penetration pressure (PP) and could be calculated based on the following equation:

PP = F_u_/S

Here, *F*_u_ is the ultimate compressive force at complete breakage of polymer and *S *is the contact area of the lower touch (32).

*Fourier transform infrared (FTIR) spectroscopy*

The chemical structure of the synthesized hydrogels was investigated by using Fourier Transform Infrared spectroscopy. The FTIR spectrum was recorded over the range of 400 - 4000 cm^-1^ by KBr pellet method using Fourier-Transform Infrared (FT-IR) spectrophotometer, (Shimadzu, FT-IR 8400S, Japan).

*Scanning electron microscopy (SEM)*

The surface morphology and porous structures of dried SPH and SPHC were examined by Scanning Electron Microscopy. Dried superporous hydrogels were fixed on a scanning electron microscope sample holder with a double sided adhesive tape and coated with a layer of gold of 150 Å for 2 min using a sputter coater (Edwards 3-150 Å, England) in a vacuum of 3x10^-1^ atm of argon gas. The sample was then examined using a scanning electron microscope (Jeol JSM T20, Tokyo, Japan).

## Results and Discussion

*Synthesis of superporous hydrogels*

**Figure 2:**
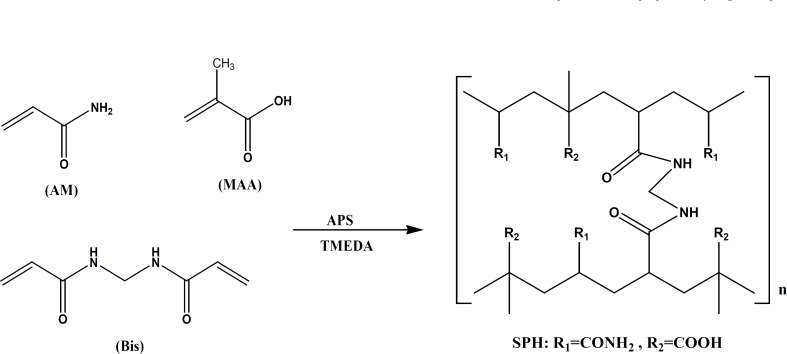
Mechanism of synthesis of pH sensitive poly (acrylamide-co-methacrylic acid) superporous hydrogel

The superporous hydrogels were synthesized using a solution polymerization technique with AM and MAA as monomers, BIS as a crosslinker, sodium bicarbonate as a blowing agent, APS as an initiator, and TEMED as a catalyst. The combination of APS and TEMED will initiate the radical polymerization. The radicals formed will then attack the double bonds of AM and MAA, and also to a less extent to the double bond of BIS. Subsequently, the double bonds will be opened and the monomers will covalently bind to each other and form a long aliphatic chain. These chains are subsequently cross-linked through the added cross-linker. The mechanism of synthesis is depicted in Figure 2. PF127 as a surfactant poly (ethylene oxide)-poly(propylene oxide)-poly(ethylene oxide) triblock copolymer, did not contribute to the chemical structure of the polymer, but could stabilize the generated foam through lowering the film-air interfacial tension and increasing the film viscosity. To obtain superporous hydrogel with well-distributed pores, gelling ought to occur when the foam took place. In superporous hydrogel composites, Ac-Di-Sol (a stabilizer) was used for introducing additional mechanical stability of the polymer through the physical entanglement of polymer chains with Ac-Di-Sol fibres.

Foaming and gelation reactions should take place simultaneously to obtain the well-established porous structures. The gelation reaction takes place only at the pH of 6-7. When gelation times are not short enough, bubbles are not stabilized but collapsed during the gelation reaction. On the other hand, the foaming reaction took place only at the acidic conditions (pH lower than 6). When sodium bicarbonate was added to the monomer solution, it was reacted with acid to start the foaming process. In the meantime, the pH of the solution was increased to 7-8 since an excess amount of sodium bicarbonate had been used. As sodium bicarbonate is decomposed to release carbon dioxide gas in acidic conditions and this decomposition reaction neutralizes the medium (increases the pH), the addition of certain amount of sodium bicarbonate eventually induced the gelation reactions at medium pH (21).

*Effect of temperature on the swelling capacity*

**Figure 3:**
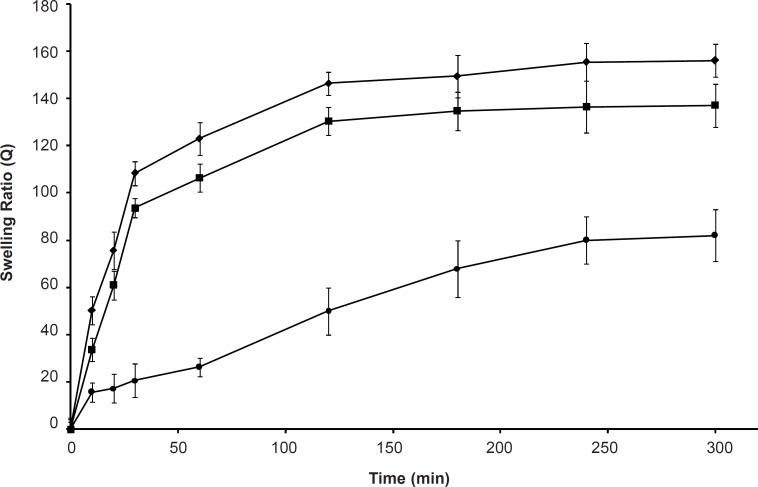
Swelling kinetics of SPHC_100_ in deionized water at (●) 20, (■) 30, and (♦) 40°C (n = 3, mean ± standard deviation).

Swelling behaviors of the SPHC_100_ at different temperatures are shown in Figure 3. As the temperature increased from 20 to 40°C, the polymer swelled faster, and the equilibrium swelling ratio was enhanced accordingly. This was due to the disentanglement of interpenetrated polymeric chains and destruction of hydrogen bonding between polymer molecules. At a higher temperature, the chain mobility was increased which facilitated the network expansion (33). Such temperature responsiveness was also attributed to the high porosity of the SPHC_100 _as the more pores would enhance the uptake of water during swelling in comparison with less porous hydrogels (34).

*Effect of the ionic strength on the swelling capacity*

**Figure 4:**
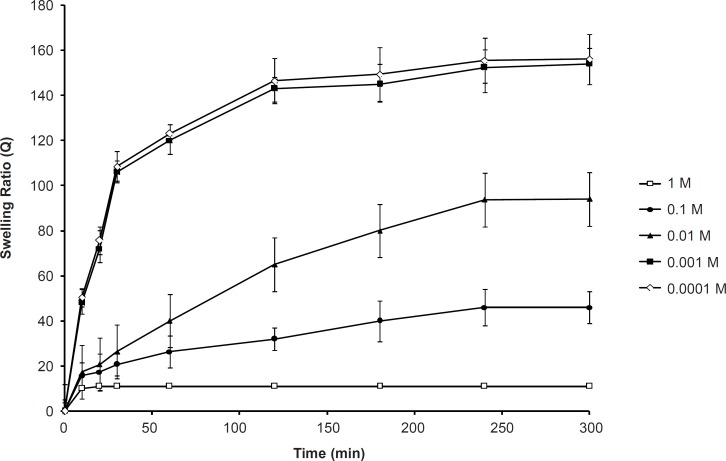
Effect of the ionic strength of swelling medium on the swelling kinetics of the SPHC_100 _(n = 3, mean ± standard deviation).

The effect of the ionic strength on the swelling capacity is shown in Figure 4. It shows that an increase in the ionic strength within the range of 0.001-1 M yields a significant decrease in the swelling ratio of SPHC_100_. When the ionic strength was less than 0.001 M, it did not affect the swelling behavior of SPHC_100._ The sensitive swelling of the anionic SPHC_100 _towards ionic strength was attributed to the change in the charge distribution on the surface of the gel network. As the concentration of cations in the swelling medium increased, a stronger ‘‘charge screening effect’’ of the additional cations was achieved, causing imperfect anion-anion electrostatic repulsion and a decreased osmotic pressure difference between the polymer network and the external solution. Therefore, swelling of SPHC_100_ was decreased. When the ionic strength was lesser than 0.001 M, the solution lacked enough ions to shield the polymer chains from one another, leading to an inappreciable influence on the swelling of the SPHC_100_.

Generally, the swelling capacity of ionic hydrogels in salt solutions is significantly decreased compared to the values in distilled water. This phenomenon can be explained on the basis of osmotic pressure developed due to the unequal distribution of ions in the medium and the polymer network. The ions attached to the polymer network are immobile and considered to be separated from the external solution through a semi-permeable membrane. When the hydrogels are placed in pure water, the maximum osmotic pressure develops and hence the maximum swelling is achieved. When the polymer is in salt solution, the development of osmotic pressure is much lower since the external solution contains Na^+^ and Cl^-^. When swollen hydrogel is put in the solutions of varying concentrations of NaCl, the presence of Na^+^ ions in the outer solution causes a decrease in the osmotic swelling pressure which operates due to the difference of counter ions in the gel phase and solution phase. With the increase in concentration of Na^+^ ions in the swelling medium, the difference between the concentration of counter ions in the gel phase and solution phase decreases, thus causing a decrease in the equilibrium water uptake of hydrogel sample. Therefore, the swelling is drastically reduced. Swelling is related to the concentration of the salt solution as follows:

Swelling = k [salt]^-n^

Here, *k *and *n *are constants for an individual superporous hydrogel. The *k *value gives the swelling at a high concentration of salt and the *n *value is a measure of salt sensitivity.

**Figure 5:**
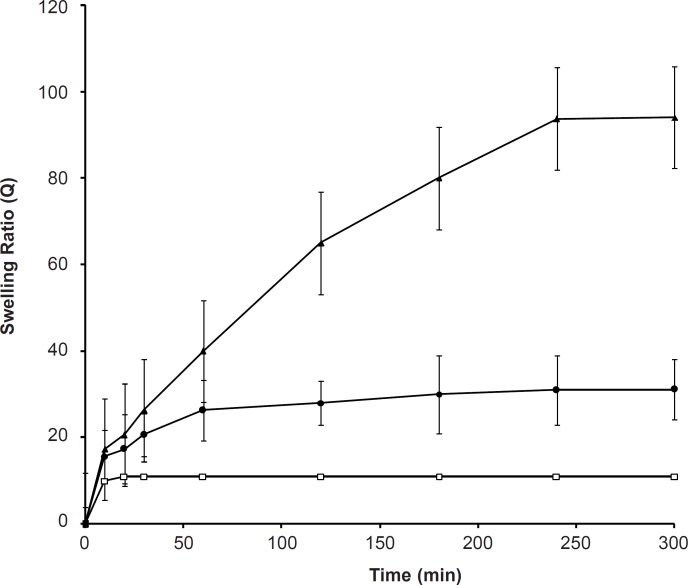
Effect of the NaCl (▲), CaCl_2_ (●) and AlCl_3_ (□) on the swelling kinetics of the SPHC_100 _(n = 3, mean ± standard deviation).

In the case of salt solutions with multivalent cations, ‘ionic crosslinking’ at the surface of the particles is another factor for the appreciable decrease in swelling capacity. According to Figure 5, the decrement in swelling amounts is strongly dependent on the type and concentration of salt added to the swelling medium. From Figure 5, it is clear that, at the same concentrations of salt solutions, the swelling ratio decreased with an increment in charge of the metal cations from Na^+^ to Ca^2+^ and Al^3+^. With increasing cation charge, the ionic strength of the medium and the degree of crosslinking are increased and the swelling capacity consequently decreases. The role of AlCl_3_ salt in decreasing the pH of solution is an additional reason for the lower water uptake of the described hydrogel (35, 36).

*Effect of pH on the swelling capacity*

**Figure 6:**
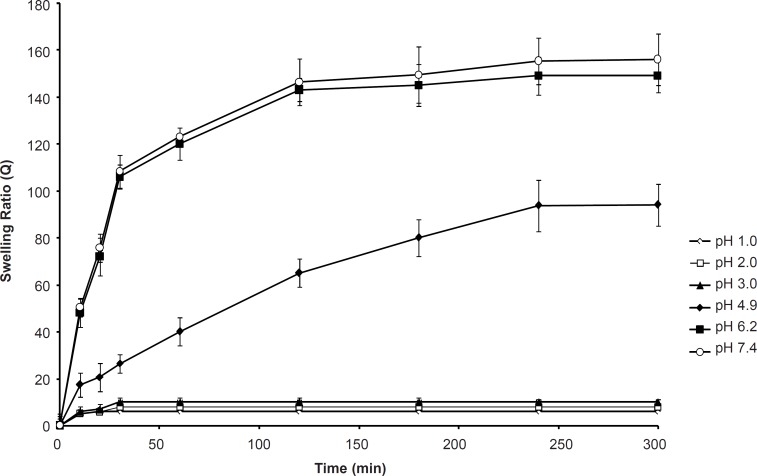
Effect of the pH of swelling medium on the swelling kinetics of the SPHC_100 _(n = 3, mean ± standard deviation).

In the design of oral delivery of peptide and protein drugs, the formulator must consider that the natural pH environment of gastrointestinal tract varies from acidic in the stomach to slightly alkaline in the intestine. Swelling of SPHC_100_ in the pH of 1.0 to 3.0 was minimal. Since the SPH and SPHC were composed of acidic groups which can dissociate or get protonated at some suitable pH of the swelling media, the degree of swelling of SPHC_100_ underwent appreciable change with external pH. Figure 6 shows the dynamic uptake of SPHC_100_ water in the solutions with pH of 1.0, 2.0, 3.0, 4.9, 6.2, and 7.4. At pH of 1.0, 2.0 and 3.0, a slight swelling capacity of the SPHC_100_ was observed due to the protonation of carboxylic groups. At a very acidic condition (pH ≤ 3.0), the carboxylic groups on poly (methacrylic acid) were converted to the protonated acid form which resulted in the decreased swelling ratio of the SPHC_100_. As pH exceeded 4.9, some carboxylate groups were ionized and the electrostatic repulsion between the carboxylate groups resulted in an enhancement of the swelling capacity (37). Moreover, the ionization also caused an increase in ion osmotic pressure. These two factors and the capillary wetting of interconnected open pores of SPHC_100_ were thus responsible for a higher degree of swelling in the medium of pH range from 4.9 to 7.4. When the pH reached 6.2, all the carboxylic groups were converted to the salt form and the maximum swelling was obtained which accounted for similar swelling behaviors at pH of 6.2 and 7.4.

*Swelling reversibility studies*

**Figure 7:**
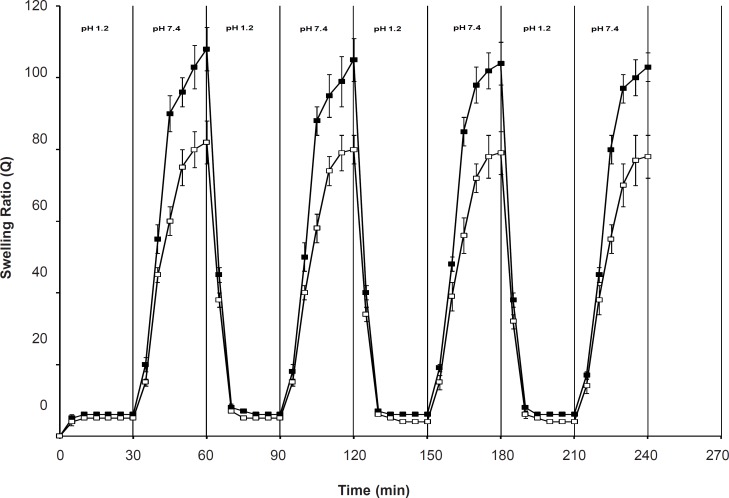
Pulsatile pH-dependent swelling behaviors of SPH (■) and SPHC_100_ (□) at 37°C with alternation of the swelling medium between HCl solution (pH = 1.2) and PBS (pH = 7.4) (n = 3, mean ± standard deviation)

Figure 7 shows the swelling reversibility of the polymers between the solutions with pH of 1.2 and pH of 7.4. Both SPH and SPHC_100_ were able to quickly absorb and deabsorb the swelling medium upon the pH change from acidic conditions to basic conditions and vice versa. The structure of the polymers with large numbers of pores connected to one another to form capillary channels was favorable for easy diffusion of the swelling medium into the polymeric matrix, thus, contributing to its quick response toward pH change. The time for swelling was longer than that for deswelling of the hydrogels and the swelling rate of SPHC_100_ in a basic external solution was slower than that of SPH. This might be due to the restricted chain mobility of poly (methacrylic acid-co-acrylamide) P (MAA-co-AM), which was anchored at several points through molecular entanglement with the Ac-Di-Sol network, since the fast pH-sensitive behavior of hydrogels was based on the freely mobile side chains (38).

*Porosity and void fraction measurements*

As shown in Table 1, the Porosity of SPHC was higher than SPH. This is due to the incorporation of the cellulosic fibres of Ac-Di-Sol within the polymer structure leading to the decrease in the occupied volume. Additionally, the void fraction of the SPHs was decreased with addition Ac-Di-Sol. These results are consistent with the decrease in swelling ratio. The decrease in void volume leaded to a decreased amount of uptake of water into the structure, causing the swelling ratio to decrease in SPHC.

*Water retention*

**Figure 8:**
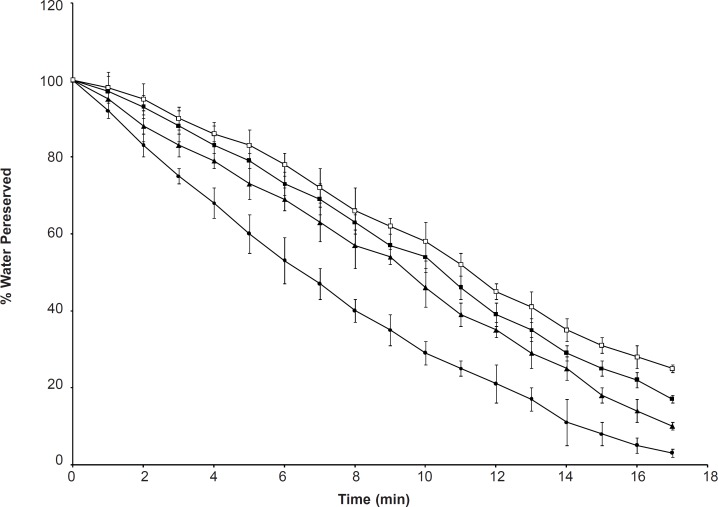
Water retention capacity of SPH (●), SPHC_100_ (▲), SPHC_200_ (■) and SPHC_400 _(□) (n = 3, mean ± standard deviation). WR_t_ is expressed as the percentage of water retained in the polymer at certain time intervals

Figure 8 shows that the polymers lost most of the water after exposure to 37^o^C for 17 h. SPHC_400_ exhibited slower water loss and a higher amount of retained water in comparison with SPHC_200_, SPHC_100 _and SPH. This suggested that a higher content of Ac-Di-Sol was favorable for the WR_t_ of SPHC. WR_t_ of the hydrogels could be attributed to the hydrophilic branch chains of P(MAA-co-AM) and Ac-Di-Sol, which created a hydrophilic environment, thus minimizing water loss from the hydrogel. The interconnected pores allowed the polymer to hold more water by capillary force. The SPHC consisting of Ac-Di-Sol had decreased polymer rigidity, thus improving the resiliency of the polymer in response to the compression and efficiently preventing water loss. Furthermore, entanglement of Ac-Di-Sol and P(MAA-co-AM) led to an enhanced amount of net points and mesh units, which favored entrapment of water. The high water affinity of Ac-Di-Sol could also account for the improved water-retention ability (39).

**Table 1:** Porosity and void fraction of SPH and SPHCs. (n = 3, mean ± standard deviation)

**Superporous Hydrogels**	**Porosity (%)**	**Void fraction (mL/g)**
**SPH**	66.3 ± 3.8	1.14 ± 0.01
**SPHC**_100_	73.2 ± 4.2	0.93 ± 0.03
**SPHC**_200_	78.6 ± 1.8	0.85 ± 0.04
**SPHC**_400_	86.4 ± 2.2	0.72 ± 0.03

*Mechanical properties*

Appropriate mechanical strength should be provided for SPHs in order of their effective application. Table 2 shows the penetration pressures of SPH and SPHCs. The results indicate that Ac-Di-Sol increased the penetration pressure values thus increasing the mechanical stability. The presence of Ac-Di-Sol increased the overall cross-linking density of the SPH composite by physical entanglement of the polymer chains with Ac-Di-Sol fibers. When Ac-Di-Sol fibers were added to the monomer solution, they swelled and absorbed the monomer solution. This resulted in physical entanglements of polymer chains through the Ac-Di-Sol fibers. This is expected to share the mechanical load between Ac-Di-Sol fiber and the polymer structure. The entanglement of P(MAA-*co*-AM) chains with Ac-Di-Sol fibers significantly improved the structural integrity of the hydrogel and decreased stress relaxation, which helped enhancing its ability to withstand the pressure.

*Fourier transform infrared (FTIR) spectroscopy*

**Figure 9:**
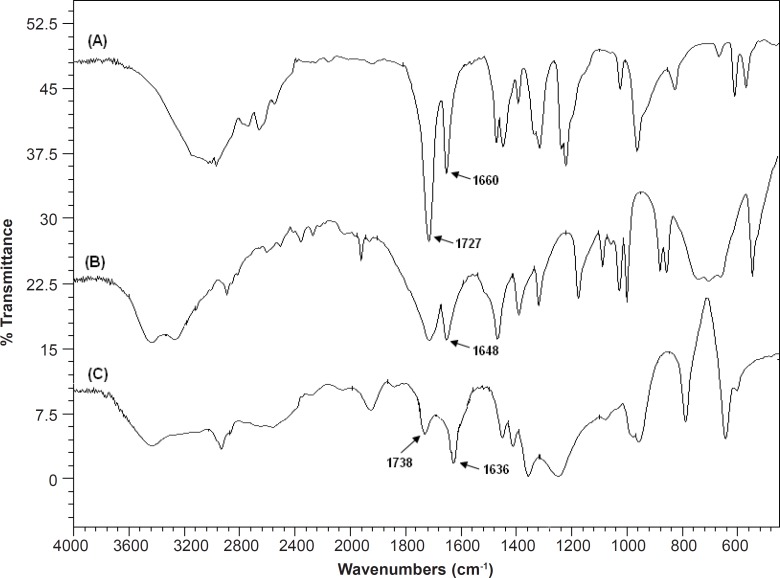
FTIR spectra of (A) Methacrylic acid (B) Acrylamide (C) SPHC100

Figure 9 shows the FTIR spectra of MAA, AM and SPHC_100_. The peaks at 1727 cm^-1 ^in the MAA spectrum and 1738 cm^-1 ^in the SPHC_100_ spectrum correspond to the stretch vibration of -COOH group, confirming the existence of the P(MAA-*co*-AM) in the SPHC_100_. Peaks at 1660 cm^-1^ in the MAA spectra, at 1648 cm^-1^ in the AM spectra and at 1636 cm^-1 ^in the SPHC_100_ spectra correspond to stretch the vibration of C=C group. However, peak intensities are significantly reduced and locations slightly deflected, which might be attributed to the molecular entanglement between P(MAA-*co*-AM) and Ac-Di-Sol (40, 41).

**Table 2:** Mechanical strength of SPH and SPHCs. (n = 3, mean ± standard deviation)

**Superporous Hydrogels**	**Porosity (%)**	**Void fraction (mL/g)**
**SPH**	66.3 ± 3.8	1.14 ± 0.01
**SPHC**_100_	73.2 ± 4.2	0.93 ± 0.03
**SPHC**_200_	78.6 ± 1.8	0.85 ± 0.04
**SPHC**_400_	86.4 ± 2.2	0.72 ± 0.03

*Scanning electron microscopy*

**Figure 10:**
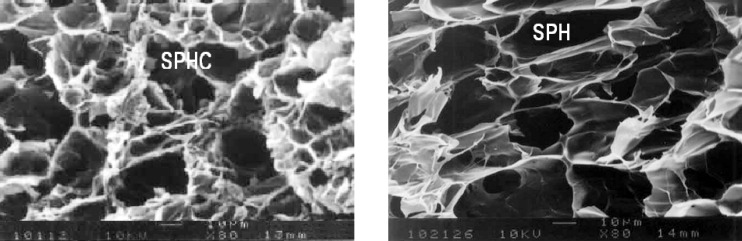
Scanning electron microscopic photograph of SPHC and SPH recorded at 80X magnification showing porous surface

The scanning electron microscopic photograph of SPH and SPHC (Figure 10) clearly shows the presence of large quantities of interconnected pores indicating a superporous structure. As compared to SPH, the SPHC also possesses large quantities of interconnected pores, indicating that the formation of composites would not destroy the superporous structure. The SPH and SPHC had high porosity and this was responsible for faster swelling of superporous hydrogels when compared to the conventional hydrogels. The SPHC had lesser pores due to the presence of Ac-Di-Sol.

## Conclusions

In the present study, the SPH and SPHC with superporous structures were synthesized through the free radical copolymerization. The polymers had fast swelling rate and high swelling ratio due to the high porosity and interconnection among some of the pores within the polymer that was approved by the SEM images. The swelling of polymers was significantly affected by the external pH, ionic strength, and temperature stimuli. The equilibrium swelling ratios exhibited high values at pH of 6.2 and 7.4 because of the ionization of the -COOH groups in the polymers. SPH showed higher swelling ratio at higher pH in comparison with SPHC. An increase in the ionic strength of the swelling medium from 0.0001 M to 1 M resulted in a decrease in the degree of swelling. The polymers exhibited responsiveness toward temperature changes on account of the increased chain mobility at a higher temperature. Because of the superporous structure, the polymers could also rapidly reach equilibrium swelling and deswelling states with pH changes between acidic and neutral conditions. A higher content of Ac-Di-Sol networks in SPHCs improved the water retention of the polymer in response to the time of exposure. As compared to SPH, the mechanical strength of SPHCs was significantly improved due to the introduction of the Ac-Di-Sol. The mechanical strength of SPHCs was increased with increasing amount of Ac-Di-Sol because of the denser network and the smaller amount of equilibrium water sorption. The unique characteristics of these superporous hydrogels open a new field of application in controlled drug delivery. As the swelling properties of both SPH and SPHC are pH-dependent, these polymers can be used as pH-sensitive drug delivery systems.
